# Crowd density estimation using deep learning for Hajj pilgrimage video analytics

**DOI:** 10.12688/f1000research.73156.1

**Published:** 2021-11-24

**Authors:** MD ROMAN BHUIYAN, Dr Junaidi Abdullah, Dr Noramiza Hashim, Fahmid Al Farid, Dr Jia Uddin, Norra Abdullah, Dr Mohd Ali Samsudin

**Affiliations:** 1 FCI, Multimedia University, Persiaran Multimedia, Cyberjaya, 63100, Malaysia; 2 Technology Studies Department, Endicott College, Woosong University, Daejeon, 100-300, South Korea; 3 WSA Venture Australia (M) Sdn Bhd, Cyberjaya, 63000, Malaysia; 4 Universiti Sains Malaysia, USM Penang, 11800, Malaysia

**Keywords:** Visual Surveillance, Density Estimation, Crowd Counting, CNN.

## Abstract

Background: This paper focuses on advances in crowd control study with an emphasis on high-density crowds, particularly Hajj crowds. Video analysis and visual surveillance have been of increasing importance in order to enhance the safety and security of pilgrimages in Makkah, Saudi Arabia. Hajj is considered to be a particularly distinctive event, with hundreds of thousands of people gathering in a small space, which does not allow a precise analysis of video footage using advanced video and computer vision algorithms. This paper aims to propose an algorithm based on a Convolutional Neural Networks model specifically for Hajj applications. Additionally, the work introduces a system for counting and then estimating the crowd density.

Methods: The model adopts an architecture which detects each person in the crowd, spots head location with a bounding box and does the counting in our own novel dataset (HAJJ-Crowd).

Results: Our algorithm outperforms the state-of-the-art method, and attains a remarkable Mean Absolute Error result of 200 (average of 82.0 improvement) and Mean Square Error of 240 (average of 135.54 improvement).

Conclusions: In our new HAJJ-Crowd dataset for evaluation and testing, we have a density map and prediction results of some standard methods.

## Introduction

Hajj has been used as an opportunity for certain rituals. The Hajj is linked to the life of the Islamic prophet Muhammad, who lived in the seventh century AD, although Muslims believe that the tradition of pilgrimage to Mecca dates all the way back to Abraham’s time. ^1^ For four to five days a year, over two million pilgrims from several parts of the world come to Mecca, where they tour the many places in Mecca and perform rituals. ^2^ Each ritual has a short but challenging path to take. The Hajj authorities have confirmed that they are having difficulties in monitoring crowd density, which can be seen from the tragedies that occurred in September 2015. ^3^ Regression-based approaches are normally used to estimate crowd density, to infer a mapping between lower-level capabilities and crowd evaluation. ^1^^,^^2^

In this paper, we propose a method for crowd analysis and density estimation using deep learning. ^2^ Our aim is to analyze the map of crowd videos and then use visualization for cross-scene crowd analysis in unseen target scenes. To do this, we must overcome the following obstacles: The challenge of prevailing multitude analysis is insufficient to help in the comparison of research into scene analysis. ^4^^–^^6^

The main contributions of this research include:

1. A methodology to accurately perform the multitude analysis from an arbitrary multitude density and arbitrary perspectives in a separate video.
2. An evaluation of interventions and a comparison of these established methods specifically for activity with recent deep CNN networks.
3. A new dataset based on Hajj pilgrimage specifically for the crowds around the Kaaba area. Crowd datasets such as Shanghai Tech, UCSD, and UCF CC 50 are available for crowd analysis research, however our dataset contains large numbers of crowds.

## Related works

Early works on the usage of detection methods in crowd counting are presented. ^7^^–^^11^ Typically, these approaches refer to an individual or head detector through a sliding picture window. Recently, many exceptional object detectors have been presented, including Region Based Convolutional Neural Networks (R-CNN), ^12^^–^^14^ YOLO ^15^ and SSD, ^16^ which can have a low precision of detection in scattered scenes. Some works such as Idrees *et al.* ^17^ and Chan *et al.* ^18^ implement regression-based approaches that learn directly from the crowd images in order to minimize these issues. They normally extract global ^19^ (texture, gradient, edge) or local characteristics ^20^ for the first step (SIFT, ^21^ LBP, ^22^ HOG, ^23^ and GLCM ^21^). Then several regression techniques such as linear regression ^24^ and Gaussian mixture regression ^25^ are employed to map the crowd counting function. These approaches manage the problems of occlusion and context disorder successfully, but spatial detail is still ignored. Thus, Lemptisky *et al.* ^26^ have developed a framework that focuses on density assessment, learning to linearly plot local features and charts. A non-linear mapping, random forest regression, which is achieved the same forest to train two separate forests, is proposed in order to reduce the challenge of studying linear mapping. ^27^ Previous heuristic models that traditionally used CNNs to estimate crowd density ^28^^–^^31^ have improved significantly compared to conventional handcrafted methods.

## Methods

**Figure 1.**
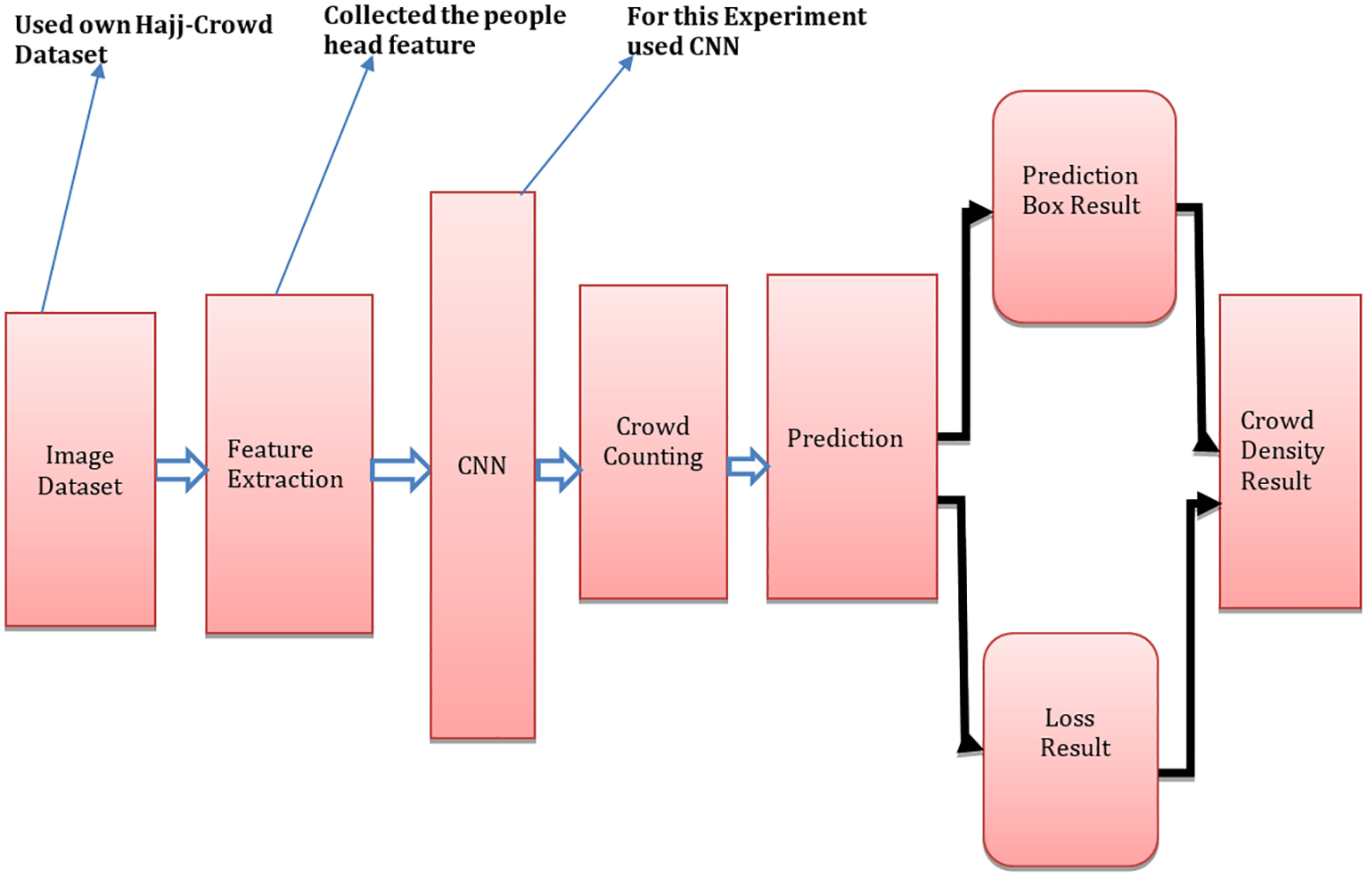
Proposed crowd counting technique based on CNN architecture.

We proposed a model that employs the state-of-the-art crowd counting algorithms used for the Hajj pilgrimage. The algorithms predicted specific regions on people’s heads for Hajj crowd images. The head size for each individual is identified using multi-stage procedures. Figure 1 shows the suggested architecture of CNNs, which is made up of three key components. The first component is the extraction of frames. To do this, we first gathered video clips of Hajj pilgrims. For this experiment we have collected video clips from YouTube using video recording software. To develop this model, we have used programming language python 3.6.15 with others libaries such as/opencv-python 3.4.11.43, NumPy 1.21.2, SciPy 1.21.2 and matplotlib 3.4.3. ^32^ We executed 30 frame extractions per second to assemble all of the footage into one clip. Feature extraction at different resolutions is the method used in spatial feature extraction. The CNN prediction map has been utilized in our proposed method. A set of multi-scale feedback reasoning networks (MSFRN) was used to route the results of mapping to the MSFRN. Results from mapping were sent to the MSFRN where information fused across the scales and predictions were formed using boxes. ^32^ Finally, crowd density results were obtained by utilizing the Non-Maximum Suppression (NMS) which uses several resolutions in combination to arrive at the accurate result. After completing the whole process we got the crowd density result. To compare with our proposed method the following existing algorithms were used. Adversarial Cross-Scale Consistency Pursuit was suggested by Zan Shen *et al.* as a new paradigm for crowd counting (density estimation) (ACSCP). A three-part Perspective Crowd Counting Network (PCC Net) has been suggested by Junyu Gao *et al.* Yuhong Li *et al.* suggested CSRNet made up of two main parts: CNN as the front-end for 2D feature extraction and a dilated CNN as the back-end. The CP-CNN developed by Vishwanath A *et al.* has four modules: the GCE, the LCE, the DME, and a Fusion-CNN (F-CNN). An image’s change in crowd density may be used to enhance the accuracy and localisation of the projected crowd count, as suggested by Deepak Babu Sam *et al.*

### Architecture of CNN layer

In addition to CNN detectors, all existing CNN-related detectors are built on a deep-backbone feature extractor network. Furthermore, it is possible that detection accuracy is linked to functionality consistency. CNN-enabled networks are often used in counting crowds, and give an approximate real time performance. ^31^ The first five CNN convolution blocks initialized using ImageNet training are the backbone network’s starting point. ^33^

### Classification of the box

Instead of making everything the same size, we used a per pixel categorization approach for scaling. The model classifies each head as part of or inside the context of one of the bounding boxes. Model scale branches generate map set ${\left\{D_{n}^{s}\right\}}_{h}^{\mathrm{nB}}=0$ , showing the confidence level for each pixel for classes of the box. The final requirement for training the model is to know the model’s users’ head sizes, which is not easily accessible and cannot be reliably inputted from typical crowd sourced databases. We created a method to help estimate head sizes in this research. We used the crowd dataset accessible point annotations to get the ground truth. People’s heads are located at certain coordinates with these annotations. Note that only quadratic boxes are regarded as box-like. However, on the whole, they are deemed experimentally effective,

$$\beta \frac{s}{b}=\left\{\begin{array}{cc} \beta \frac{s+1}{\mathrm{ns}} & \text{if}s<\mathrm{ns}-1\\ 1+\left(b-1\right)y^{s}, & \text{Otherwise} \end{array}\right.$$ (1)

In choosing the Box U+03B2 ( *s*)/ *b* *s* for each scale, a popular approach is used. At the maximum resolution scale ( *s* = *ns* U+2212 1), the initial box size ( *b* = 1) is often set at one, which increases the ability to handle the extremely congested density. The standard size of increase values on different scales are the *y* = 4, 2, 1, 1 definition. Please note that at high-level (0.5 and 0.25), in which coarse resolution is appropriate (as shown by Figure 1), boxes of better sizes include those of low resolution (0.16 and 0.25). ^33^

### Count of heads

For testing the model in Figure 1, the predictive fusion procedure is utilized in place. The multi-resolution prediction is made across all branches of the picture pipeline. Using these prediction charts, we can anticipate that the locations of the boxes are linearly scaled from the resolution of the input. When the present NMS is in place, then it is used to prevent multi-threshold mixing.

### Data collection

The HAJJ-crowd dataset was collected from live television broadcasts via YouTube of the Mecca Hajj 2019. All of the images depict pilgrims performing tawaf around the magnificent kaaba. Tawaf involves walking around the Kabba seven times. The moving process begins in the opposite direction of the clock. The video frames have been extracted and saved as.jpg files for future examination. The dataset contains a total of 1500 crowd images. As a result, 1500 images and ten film sequences are captured in several populous areas surrounding Kaaba (Tawaf region), with some typical crowd scenarios, such as touching a black stone in the Kaaba region and tossing a stone into the Mina region. All images have a resolution 1280 × 720 HD and videos have a resolution 1080p.

### Annotation technique

We used python 3.6.15 and opencv-python 3.4.11.43 as an annotation tool to easily annotate head positions in the crowds. The process involved two types of labelling: point and bounding box. During the annotation process, the head is freely zoomed in/out, split into a maximum of 3 × 3 tiny patches, allowing annotators to mark a head in 5 sizes: 2x (x = 0,1,2,3,4) times the original image size.

### Experimental design

Firstly, we gathered all images of size 1280 × 720 pixels. Then we applied a profound learning method to improve the CNN and obtain the best outcomes. Training and analysis was done using the pytorch 1.9.1 framework and operating system Ubuntu 18.04.6 LTS deep learning packages on NVIDIA GEFORCE GTX 1660Ti GPU. For profound learning, we utilized packages such as opencv-python 3.4.11.43, NumPy 1.21.2, SciPy 1.21.2, matplotlib 3.4.3.

### Experimental analysis

The HAJJ-crowd data collection consisted of three sections, the examination, validation and training. The count accuracy which is the Mean Absolute Error (MAE) and Mean Squared Error (MSE) should be measured in two measurements. The equations are shown below:

$$\text{MAE}=\frac{1}{N}\sum_{i=1}^{N}\left|\mathrm{yi}-y^{\prime }i\right|$$ (2)

$$\text{MSE}=\sqrt{\frac{1}{\;N}\sum_{i=1}^{N}{\left|y_{i}-y_{i}\right|}^{2}}$$ (3)

In this scenario, *N* is assumed to be the test sample, *y*_*i*_ is regarded as the count mark, whereas *y*′ _*i*_ is the approximation count sample. For each set of persons, the preceding group consists of (0), (0, 1000) (1000, 2000), (2000, 3000). In accordance with the annotated number and quality of the image, each image is allocated an attributing label. In the test set, MAE and MSE are applied for the matching samples in a particular viewpoint for each class. For example, the luminescence attribute calculates average MSE and MAE figures based on two categories that demonstrate the counting models’ sensitivity to luminescence variation.

## Results analysis

**Figure 2.**
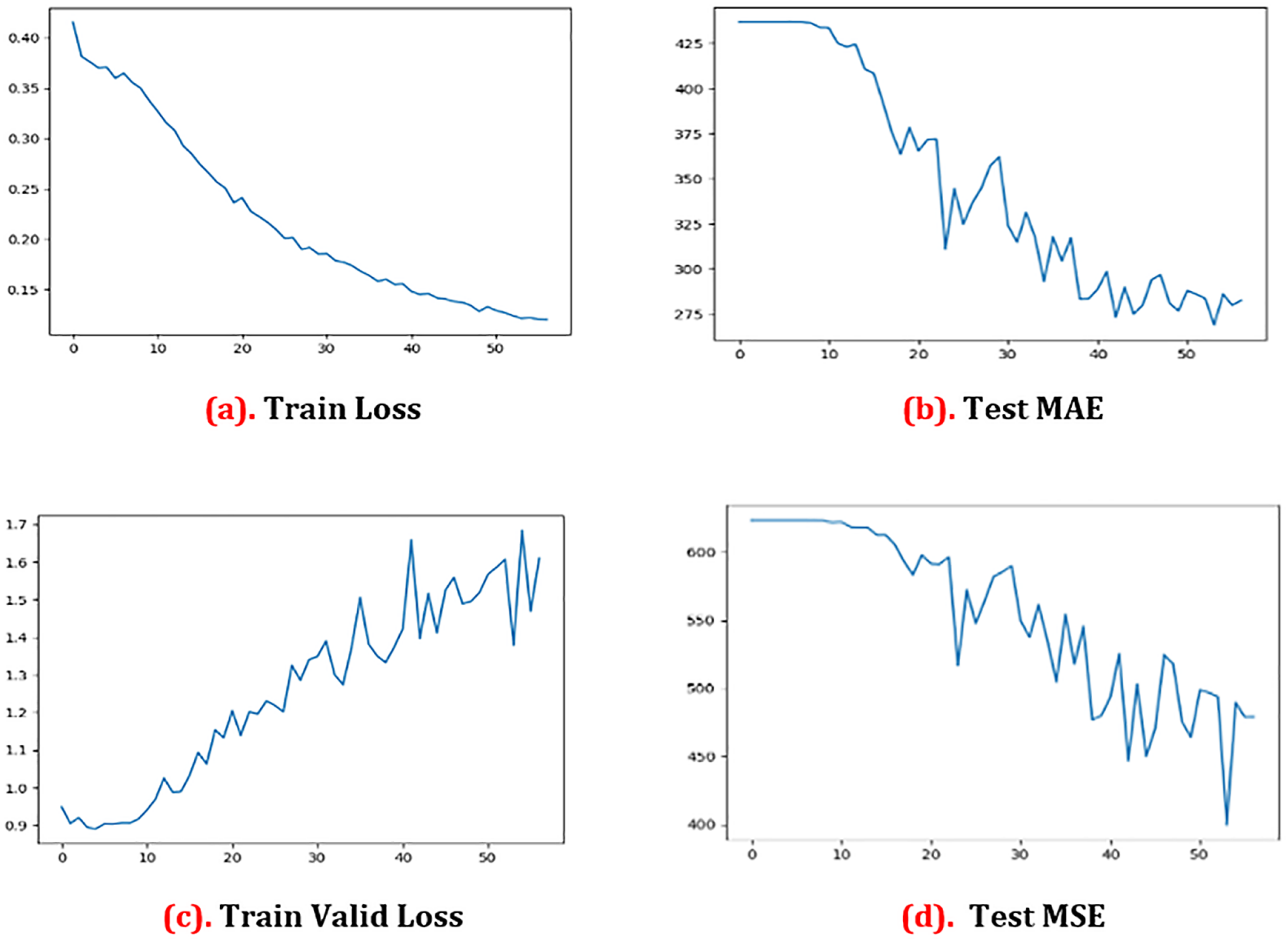
Results analysis graph. MAE = mean absolute error; MSE = mean squared error.

Figure 2(a) and Figure 2(c) indicate clearly that there is no significant change in the loss of pixels from zero to ten epochs, whereas there is a ten pixel loss from ten to 20 epochs. However, the pixel loss between 20 and 30 epochs keeps increasing, up to 40–52 epochs. At the end, the pixel loss is 15.0 at 52 epochs. We may get genuine training loss from this experiment. More than anything, the legitimate pixel loss in tests is 17 at 40 epochs and 14 at 52 epochs. At the same time, based on the preceding equation, we computed the MAE test. We have computed the valid MAE test loss and the valid MAE test that is shown in Figure 2(b) and Figure 2(d). For the MAE test, we found that the error is over 600 when the epoch is zero. We saw the error coming down to 200.0 after 52 epochs. In the Test MSE, we saw the error is over 425 if the epoch is zero. After that, we saw that the error came down to 240.0, after 52 epochs. Figure 2 shows the graphical representations of the results.

### Proposed method comparison with state-of-the-art methods

The HAJJ-crowd dataset contains a large number of crowds as well as a density collection. It contains 1050 training images and 450 testing images with the same resolution of 1280 × 720 pixels. The mainstream UCF CC 50 dataset are compared with the most advanced non-defined approaches ^34^^–^^38^ in terms of the MAE and MSE. Our method and dataset outperforms the state-of-the-art methods, and attains a remarkable MAE result of: 200.0 (Average of 82.0 points improvement) and MSE of 240.0 (Average of 135.54 points improvement). Table 1 shows the comparison with state-of-the-art methods.

**Table 1.** Error estimation on UCF CC 50 dataset. MAE = mean absolute error; MSE = mean squared error.

Method	MAE	MSE
ACSCP ^34^	291.0	404.6
PCC Net ^35^	240.0	315.5
Switching-CNN ^36^	318.1	439.2
CP-CNN ^37^	295.8	320.9
CSRNet ^38^	266.1	397.5
**Proposed method**	**200.0**	**240.0**

## Conclusions

This paper provides a new approach for crowd density estimation using a convolutional neural network. A multi-column structure of high-level feedback processing that addresses the problems in large crowds is the proposed model of the convolutional neural network. The proposed model can recognize moving crowds, which leads to improved performance. We found that crowd analysis prior to crowd counting has significantly boosted the efficiency of counting for extremely dense crowd scenarios.

## Data availability

### Underlying data

Due to the ethical and copyright limitations around social media data, the underlying data for this study cannot be disclosed. The original dataset contains a total of 1500 images, all of which were collected from the Mecca Hajj 2019. The dataset contains three classes of crowd density around tawaf area. The Methods section offers extensive information that will enable the research to be replicated. If you have any questions concerning the approach, please contact the corresponding author.

### Software availability

Software available from: https://github.com/romanbhuiyan/CrowdCounting.

Archived source code at time of publication: https://doi.org/10.5281/zenodo.5635486. ^32^

License: https://opensource.org/licenses/gpl-licenseGPL.

## Author contributions

R.B. developed the experimental model, structure of the manuscript, performance evaluation and wrote the preliminary draft. J.A. helped to fix the error code, checked the labelled data and results as well as reviewed the full paper. N.H. gave some important feedback on this paper. F.F. helped with the structured full paper revision. J.U. helped format the full paper. N.A. checked the revised version and added a few paragraphs to the full article. M.A.S. helped with the paper organization. All authors discussed the results and contributed to the final manuscript.

## References

[1] Armstrong K : Islam: A short history. modern library chronicles (revised updated ed.). *Modern Library.* 2020; (pp.10–12). 0-8129-6618-X.

[2] Alazbah A Zafar B : Pilgrimage (hajj) crowd management using agent-based method. *International Journal on Foundations of Computer Science & Technology.* 2019;09(1):01–17. 10.5121/ijfcst.2019.9101

[3] S. and agencies : A history of hajj tragedies.). *The Guardian.* Jan. 13, 2006. (accessed Aug. 31, 2021). Reference Source

[4] Chen K Gong S Xiang T : Cumulative attribute space for age and crowd density estimation. *Proceedings of the IEEE Conference on Computer Vision and Pattern Recognition.* 2013; pages2467–2474.

[5] Chen K Loy CC Gong S : Feature mining for localised crowd counting. *Bmvc.* 2012;1:3.

[6] Chan AB Liang Z-SJ Vasconcelos N : Privacy preserving crowd monitoring: Counting people without people models or tracking. *2008 IEEE Conference on Computer Vision and Pattern Recognition.* IEEE;2008; pages1–7.

[7] Idrees H Saleemi I Seibert C : Multi-source multi-scale counting in extremely dense crowd images. *Proceedings of the IEEE Conference on Computer Vision and Pattern Recognition.* 2013; pages2547–2554.

[8] Topkaya IS Erdogan H Porikli F : Counting people by clustering person detector outputs. *2014 11th IEEE International Conference on Advanced Video and Signal Based Surveillance (AVSS).* IEEE;2014; pages313–318.

[9] Li M Zhang Z Huang K Tan T : Estimating the number of people in crowded scenes by mid based foreground segmentation and head-shoulder detection. *2008 19th International Conference on Pattern Recognition.* IEEE;2008; pages1–4.

[10] Wang L Lisheng X Yang M-H : Pedestrian detection in crowded scenes via scale and occlusion analysis. *2016 IEEE International Conference on Image Processing (ICIP).* IEEE;2016; pages1210–1214.

[11] Enzweiler M Gavrila DM : Monocular pedestrian detection: Survey and experiments. *IEEE Transactions on Pattern Analysis and Machine Intelligence.* 2008;31(12):2179–2195. 10.1109/TPAMI.2008.260 19834140

[12] Girshick R : Fast r-cnn. *Proceedings of the IEEE International Conference on Computer Vision.* 2015; pages1440–1448.

[13] Ren S He K Girshick R : Faster r-cnn: Towards real-time object detection with region proposal networks. *Adv. Neural Inf. Process. Syst.* 2015;28:91–99. 10.1109/TPAMI.2016.2577031 27295650

[14] Minghu W Yue H Wang J : Object detection based on rgc mask r-cnn. *IET Image Processing.* 2020;14(8):1502–1508. 10.1049/iet-ipr.2019.0057

[15] Redmon J Divvala S Girshick R : You only look once: Unified, real-time object detection. *Proceedings of the IEEE Conference on Computer Vision and Pattern Recognition.* 2016; pages779–788.

[16] Liu W Anguelov D Erhan D : Ssd: Single shot multibox detector. *European Conference on Computer Vision.* Springer;2016; pages21–37. 10.1007/978-3-319-46448-0_2

[17] Idrees H Saleemi I Seibert C : Multi-source multi-scale counting in extremely dense crowd images. *Proceedings of the IEEE Conference on Computer Vision and Pattern Recognition.* 2013; pages2547–2554.

[18] Chan AB Vasconcelos N : Counting people with low-level features and bayesian regression. *IEEE Transactions on Image Processing.* 2011;21(4):2160–2177. 10.1109/TIP.2011.2172800 22020684

[19] Chen K Loy CC Gong S : Feature mining for localised crowd counting. *Bmvc.* 2012;1:3.

[20] Ryan D Denman S Fookes C : Crowd counting using multiple local features. *2009 Digital Image Computing: Techniques and Applications.* IEEE;2009; pages81–88.

[21] Lowe DG : Object recognition from local scale-invariant features. *Proceedings of the Seventh IEEE International Conference on Computer Vision.* IEEE;1999; volume2: pages1150–1157.

[22] Ojala T Pietikäinen M Mäenpää T : Gray scale and rotation invariant texture classification with local binary patterns. *European Conference on Computer Vision.* Springer;2000; pages404–420.

[23] Surasak T Takahiro I Cheng C-h : Histogram of oriented gradients for human detection in video. *2018 5th International Conference on Business and Industrial Research (ICBIR).* IEEE;2018; pages172–176.

[24] Paragios N Ramesh V : A mrf-based approach for real-time subway monitoring. *Proceedings of the 2001 IEEE Computer Society Conference on Computer Vision and Pattern Recognition. CVPR 2001.* IEEE;2001; volume1: pagesI–I.

[25] Tian Y Sigal L Badino H : Latent gaussian mixture regression for human pose estimation. *Asian Conference on Computer Vision.* Springer;2010; pages679–690.

[26] Li H Zahr M : Learning to recognize objects in images. *Trends Cogn. Sci.* 2012;3(3):1–5. 10.1016/s1364-6613(98)01261-3 10234223

[27] Pham V-Q Kozakaya T Yamaguchi O : Count forest: Co-voting uncertain number of targets using random forest for crowd density estimation. *Proceedings of the IEEE International Conference on Computer Vision.* 2015; pages3253–3261.

[28] Wang C Zhang H Yang L : Deep people counting in extremely dense crowds. *Proceedings of the 23rd ACM International Conference on Multimedia.* 2015; pages1299–1302.

[29] Min F Pei X Li X : Fast crowd density estimation with convolutional neural networks. *Engineering Applications of Artificial Intelligence.* 2015;43:81–88. 10.1016/j.engappai.2015.04.006

[30] Zhang C Li H Wang X : Cross-scene crowd counting via deep convolutional neural networks. *Proceedings of the IEEE Conference on Computer Vision and Pattern Recognition.* 2015; pages833–841.

[31] Walach E Wolf L : Learning to count with cnn boosting. *European Conference on Computer Vision.* Springer;2016; pages660–676.

[32] Bhuiyan MR Abdullah J Hashim N : Crowd density estimation using deep learning for hajj pilgrimage video analytics. 2021. 10.5281/zenodo.5635486 PMC8787568 35136582

[33] Sam DB Peri SV Sundararaman MN : Locate, size and count: Accurately resolving people in dense crowds via detection. *IEEE Transactions on Pattern Analysis and Machine Intelligence.* 2020. 10.1109/TPAMI.2020.2974830 32086197

[34] Shen Z Yi X Ni B : Crowd counting via adversarial cross-scale consistency pursuit. *Proceedings of the IEEE Conference on Computer Vision and Pattern Recognition.* 2018; pages5245–5254.

[35] Gao J Wang Q Li X : Pcc net: Perspective crowd counting via spatial convolutional network. *IEEE Transactions on Circuits and Systems for Video Technology.* 2019;30(10):3486–3498.

[36] Sam DB Surya S Venkatesh Babu R : Switching convolutional neural network for crowd counting. *Proceedings of the IEEE Conference on Computer Vision and Pattern Recognition.* 2017; pages5744–5752.

[37] Sindagi VA Patel VM : Generating high-quality crowd density maps using contextual pyramid cnns. *Proceedings of the IEEE International Conference on Computer Vision.* 2017; pages1861–1870.

[38] Li Y Zhang X Chen D : Csrnet: Dilated convolutional neural networks for understanding the highly congested scenes. *Proceedings of the IEEE Conference on Computer Vision and Pattern Recognition.* 2018; pages1091–1100.

