# Supplementary material for: Crowd density estimation using deep learning for Hajj pilgrimage video analytics
Source: F1000Res. 2022 Jan 14;10:1190. Originally published 2021 Nov 24. [Version 2] doi: 10.12688/f1000research.73156.2 (PMC8787568; doi:10.12688/f1000research.73156.2)
Supplement: Peer review history

# Peer review: Reviewer response for version 2

**DOI:** 10.5256/f1000research.119864.r119888

**Published:** 2022-01-28

**Authors:** Mohamed Uvaze Ahamed

**Affiliations:** 1 Westminster International University in Tashkent, Tashkent, Uzbekistan

No Further comments to make. As per the suggestion, the authors have addressed all my concerns and I think the paper can be indexed in its current version.

Is the work clearly and accurately presented and does it cite the current literature?

Yes

If applicable, is the statistical analysis and its interpretation appropriate?

Yes

Are all the source data underlying the results available to ensure full reproducibility?

Partly

Is the study design appropriate and is the work technically sound?

Yes

Are the conclusions drawn adequately supported by the results?

Yes

Are sufficient details of methods and analysis provided to allow replication by others?

No

Reviewer Expertise:

Computer Vision, Image Processing and Machine Learning

I confirm that I have read this submission and believe that I have an appropriate level of expertise to confirm that it is of an acceptable scientific standard.

# Peer review: Reviewer response for version 2

**DOI:** 10.5256/f1000research.119864.r119887

**Published:** 2022-01-24

**Authors:** Saravana Balaji B

**Affiliations:** 1 Department of Information Technology, Lebanese French University, Erbil, Iraq

The authors revised the article satisfactorily. Revision accepted.

Is the work clearly and accurately presented and does it cite the current literature?

Yes

If applicable, is the statistical analysis and its interpretation appropriate?

Partly

Are all the source data underlying the results available to ensure full reproducibility?

Partly

Is the study design appropriate and is the work technically sound?

Partly

Are the conclusions drawn adequately supported by the results?

Partly

Are sufficient details of methods and analysis provided to allow replication by others?

Yes

Reviewer Expertise:

Machine Learning, Deep Learning, Cloud Computing.

I confirm that I have read this submission and believe that I have an appropriate level of expertise to confirm that it is of an acceptable scientific standard.

# Peer review: Reviewer response for version 2

**DOI:** 10.5256/f1000research.119864.r119886

**Published:** 2022-01-24

**Authors:** Md Junayed Hasan

**Affiliations:** 1 Department of Electrical, Electronics and Computer Engineering, University of Ulsan, Ulsan, South Korea

I am satisfied with the answers given by the authors. Therefore, I think this paper can be indexed and is suitable for the readers. 

 The most interesting part of the paper is Hajj crowds. The authors came up with some really necessary topics.

Is the work clearly and accurately presented and does it cite the current literature?

Yes

If applicable, is the statistical analysis and its interpretation appropriate?

Partly

Are all the source data underlying the results available to ensure full reproducibility?

Partly

Is the study design appropriate and is the work technically sound?

Yes

Are the conclusions drawn adequately supported by the results?

Yes

Are sufficient details of methods and analysis provided to allow replication by others?

Partly

Reviewer Expertise:

Machine Learning, Deep Learning, Data Science, Computer Vision, Fault Diagnosis

I confirm that I have read this submission and believe that I have an appropriate level of expertise to confirm that it is of an acceptable scientific standard.

# Peer review: Reviewer response for version 1

**DOI:** 10.5256/f1000research.76787.r101072

**Published:** 2021-12-20

**Authors:** Saravana Balaji B

**Affiliations:** 1 Department of Information Technology, Lebanese French University, Erbil, Iraq

The introduction section is brief: highlight the need for density estimation and the current issues in density estimation. Also, emphasize the short note about the proposed method.

 The related work section should group the works technically and highlight the drawbacks.

 The classification of heads from the images is not clearly defined mathematically; explain it.

 The annotation process should be explained clearly: point and bounding box - define them.

 The abstract should be rewritten, "This paper aims to propose an algorithm" - this statement is not correct.

 The conclusion also does not emphasize the technical solution and the findings, rewrite accordingly.

Is the work clearly and accurately presented and does it cite the current literature?

Yes

If applicable, is the statistical analysis and its interpretation appropriate?

Partly

Are all the source data underlying the results available to ensure full reproducibility?

Partly

Is the study design appropriate and is the work technically sound?

Partly

Are the conclusions drawn adequately supported by the results?

Partly

Are sufficient details of methods and analysis provided to allow replication by others?

Yes

Reviewer Expertise:

Machine Learning, Deep Learning, Cloud Computing.

I confirm that I have read this submission and believe that I have an appropriate level of expertise to confirm that it is of an acceptable scientific standard, however I have significant reservations, as outlined above.

# Response

**Published:** 2022-01-10

**Authors:** MD ROMAN BHUIYAN

**Affiliations:** Multimedia University, Malaysia, Malaysia

1. The introduction section is brief: highlight the need for density estimation and the current issues in density estimation. Also, emphasize the short note about the proposed method.

 Ans: The benefit of the Convolutional Neural Network (CNN) model is that it is superior than handcrafted features in identifying crowd-specific characteristics. We propose a framework for crowd counting based on convolutional neural networks (CNNs) in this study.

 2. The related work section should group the works technically and highlight the draw backs.

 Ans: Considering the drawbacks of these conventional methods we have employed improved CNN.

 3. The classification of heads from the images is not clearly defined mathematically; explain it.

 Ans: It is situated approximately in the center of the head, though it may vary drastically depending on the number of people. The same applies to scale, since it not only indicates the scale of each person in the crowd, but also shows scale in the form of annotation points. Assuming a homogeneous density of the crowd, the space between two nearby people may represent the size of the box, depending on the dimensions of the crowd. Know that only quadratic boxes are regarded as box-like. In simpler words, a given head size is equivalent to the length of the neighbor closest to it. It is right to use these boxes for crowds of medium to large sizes, but for those with sparse populations with far closest neighbors, these box dimensions may be wrong. However, on the whole, they are deemed experimentally effective, providing an accurate distribution of head sizes throughout a broad range of densities.

 4. The annotation process should be explained clearly: point and bounding box - define them.

 Ans: In this study, we developed a technique for estimating head sizes. To get the ground truth, we utilized available point annotations from the crowd dataset. With these annotations, the heads of individuals are positioned at certain locations. It is worth noting that only quadratic boxes are considered box-like. It is located about in the middle of the head, but this might vary significantly depending on the population. The same holds true for scale, which not only represents the size of each individual in the crowd but also displays scale in the form of annotation points.

 5. The abstract should be rewritten, "This paper aims to propose an algorithm" - this statement is not correct.

 Ans: This research proposes an algorithm based on a Convolutional Neural Networks model specifically for Hajj applications.

 6. The conclusion also does not emphasize the technical solution and the findings, rewrite accordingly.

 Ans: The proposed method outperforms the state-of-the-art method, with a Mean Absolute Error of 200 and a Mean Square Error of 240.

# Peer review: Reviewer response for version 1

**DOI:** 10.5256/f1000research.76787.r101069

**Published:** 2021-12-06

**Authors:** Mohamed Uvaze Ahamed

**Affiliations:** 1 Westminster International University in Tashkent, Tashkent, Uzbekistan

The authors proposed a model for crowd density estimation using a convolutional neural network. Overall, the article is a clear, concise, and well-written manuscript. Though, the work has been well presented with neat technical flow, there are certain clarifications that need to be addressed by the author.

 The following comments are the possible doubts/suggestions that need to be worked carefully for the betterment of the overall article:

1. In the Methods section, the authors wrote the following statement:
  “Figure 1 shows the suggested architecture of CNNs, which is made up of three key components”.
  But actually Figure 1 shows the proposed model. Also, it doesn’t explain the architecture of CNN or DNN.
2. Due to ethical issues, the authors haven’t supplied the exact dataset. Also, the authors described in Methods section that they have collected the data from YouTube videos. Probably, the authors could provide the video link in the reference, so that the real complexity in the video could be understandable.
3. Also author need to clarify the necessity of using MAE and MSE as their performance analysis.

Is the work clearly and accurately presented and does it cite the current literature?

Yes

If applicable, is the statistical analysis and its interpretation appropriate?

Yes

Are all the source data underlying the results available to ensure full reproducibility?

Partly

Is the study design appropriate and is the work technically sound?

Yes

Are the conclusions drawn adequately supported by the results?

Yes

Are sufficient details of methods and analysis provided to allow replication by others?

No

Reviewer Expertise:

Computer Vision, Image Processing and Machine Learning

I confirm that I have read this submission and believe that I have an appropriate level of expertise to confirm that it is of an acceptable scientific standard, however I have significant reservations, as outlined above.

# Response

**Published:** 2022-01-10

**Authors:** MD ROMAN BHUIYAN

**Affiliations:** Multimedia University, Malaysia, Malaysia

1. In the Methods section, the authors wrote the following statement: “Figure 1 shows the suggested architecture of CNNs, which is made up of three key components”. But actually Figure 1 shows the proposed model. Also, it doesn’t explain the architecture of CNN or DNN.

 Ans: Typically, a CNN design consists of a single input layer, many convolutional and pooling layers, numerous fully connected layers, and a final output layer for automating the feature extraction process. As input, an RGB crowd image of 224 by 224 pixels is accepted, with data down sampling in each block for maximum pooling. Except for the last blocks, which are copied by the following blocks, every block on the network branches. A resolution of 0.5, 0.25, 0.125, and 0.166 is utilized to generate feature maps when using cloned blocks.

 2. Due to ethical issues, the authors haven’t supplied the exact dataset. Also, the authors described in Methods section that they have collected the data from YouTube videos. Probably, the authors could provide the video link in the reference, so that the real complexity in the video could be understandable.

 Ans: Ma J: *عبدالودود حنيف ماتيسر من سورة يوسف والرعد وإبراهيم*. Available at: https://www.youtube.com/watch?v=oxKSe6bFY_E, 2019 (Accessed: 28 December 2021).

 3. Also author need to clarify the necessity of using MAE and MSE as their performance analysis.

 Ans: MSE is calculated as follows, which makes mathematical operations easier than with a non-differentiable function such as MAE. That is why MAE and MSE are very important for the performance analysis in our experiment.

# Peer review: Reviewer response for version 1

**DOI:** 10.5256/f1000research.76787.r101065

**Published:** 2021-11-29

**Authors:** Md Junayed Hasan

**Affiliations:** 1 Department of Electrical, Electronics and Computer Engineering, University of Ulsan, Ulsan, South Korea

This paper focuses on recent developments in crowd control research, with a focus on high-density crowds, particularly those attending the Hajj. In order to improve the safety and security of pilgrimages in Makkah, Saudi Arabia, video analysis and visual surveillance have become increasingly important. The Hajj is a unique event in that it brings together hundreds of thousands of people in a limited space, making it difficult to analyze video material using advanced video and computer vision algorithms. The goal of this paper is to present a Hajj-specific method based on a Convolutional Neural Networks model. The work also proposes a method for counting and then estimating crowd density.

 A very unique work indeed. However, few suggestions to the authors.

1. The details of train, test, and validation is not clear from the manuscript. How much data you have actually used to perform this test? Train, test, and Validation – specifically.
2. Please mention about the cross-fold validation. How much fold you have used?
3. How did you tune your hyperparameters?
4. For the result section, why did you only use MSE and MAE. What about Precision, Recall, and F1? Unless the authors include these results, it is not justifiable.

Is the work clearly and accurately presented and does it cite the current literature?

Yes

If applicable, is the statistical analysis and its interpretation appropriate?

Partly

Are all the source data underlying the results available to ensure full reproducibility?

Partly

Is the study design appropriate and is the work technically sound?

Yes

Are the conclusions drawn adequately supported by the results?

Yes

Are sufficient details of methods and analysis provided to allow replication by others?

Partly

Reviewer Expertise:

Machine Learning, Deep Learning, Data Science, Computer Vision, Fault Diagnosis

I confirm that I have read this submission and believe that I have an appropriate level of expertise to confirm that it is of an acceptable scientific standard, however I have significant reservations, as outlined above.

# Response

**Published:** 2022-01-10

**Authors:** MD ROMAN BHUIYAN

**Affiliations:** Multimedia University, Malaysia, Malaysia

1. The details of train, test, and validation is not clear from the manuscript. How much data you have actually used to perform this test? Train, test, and Validation – specifically.

 Ans: For our Hajj-Crowd dataset, we have used 80% data for training and 20% data for testing and we could successfully validate 90% data.

 2. Please mention about the cross-fold validation. How much fold you have used?

 Ans: For our experiment, we have used three fold cross validation. 

 3. How did you tune your hyper parameters?

 Ans: We have defined the range of possible values for all hyperparameters and a method for sampling hyperparameter values as well as an evaluative criteria and a cross-validation method.

 In general, this process includes:

1. Define a model.
2. Define the range of possible values for all hyperparameters.
3. Define a method for sampling hyperparameter values.
4. Define an evaluative criteria to judge the model.
5. Define a cross-validation method.

4. For the result section, why did you only use MSE and MAE. What about Precision, Recall, and F1? Unless the authors include these results, it is not justifiable.

 Ans: To the best of our knowledge, as we are doing crowd counting here, that is why it is not mandatory to include Precision, Recall, and F1 score.
